# UltrARsound: in situ visualization of live ultrasound images using HoloLens 2

**DOI:** 10.1007/s11548-022-02695-z

**Published:** 2022-07-01

**Authors:** Felix von Haxthausen, Rafael Moreta-Martinez, Alicia Pose Díez de la Lastra, Javier Pascau, Floris Ernst

**Affiliations:** 1 Departamento de Bioingeniería e Ingeniería Aeroespacial, Universidad Carlos III de Madrid, Avda. Universidad 30, 28911 Leganés, Spain; 2 Institute for Robotics and Cognitive Systems, University of Lübeck, Ratzeburger Allee 160, 23562 Lübeck, Schleswig-Holstein Germany

**Keywords:** Augmented reality, Tracking, Ultrasound guidance, Retroreflective spheres

## Abstract

****Purpose**:**

Augmented Reality (AR) has the potential to simplify ultrasound (US) examinations which usually require a skilled and experienced sonographer to mentally align narrow 2D cross-sectional US images in the 3D anatomy of the patient. This work describes and evaluates a novel approach to track retroreflective spheres attached to the US probe using an inside-out technique with the AR glasses HoloLens 2. Finally, live US images are displayed in situ on the imaged anatomy.

****Methods**:**

The Unity application UltrARsound performs spatial tracking of the US probe and attached retroreflective markers using the depth camera integrated into the AR glasses—thus eliminating the need for an external tracking system. Additionally, a Kalman filter is implemented to improve the noisy measurements of the camera. US images are streamed wirelessly via the PLUS toolkit to HoloLens 2. The technical evaluation comprises static and dynamic tracking accuracy, frequency and latency of displayed images.

****Results**:**

Tracking is performed with a median accuracy of 1.98 mm/1.81$^\circ$ for the static setting when using the Kalman filter. In a dynamic scenario, the median error was 2.81 mm/1.70$^\circ$. The tracking frequency is currently limited to 20 Hz. 83% of the displayed US images had a latency lower than 16 ms.

****Conclusions**:**

In this work, we showed that spatial tracking of retroreflective spheres with the depth camera of HoloLens 2 is feasible, achieving a promising accuracy for in situ visualization of live US images. For tracking, no additional hardware nor modifications to HoloLens 2 are required making it a cheap and easy-to-use approach. Moreover, a minimal latency of displayed images enables a real-time perception for the sonographer.

## Introduction

Ultrasound (US) as a radiation-free, portable, widely available, and real-time capable imaging modality has become one of the core diagnostic tools for a variety of diseases. It has also proved application possibilities in therapeutic areas ranging from bone fracture healing to cancer treatment [1]. In addition, general practitioners, usually less experienced with this imaging modality, are increasingly using US in their daily practice [2].

However, US requires an experienced and skilled sonographer mentally aligning the narrow cross-sectional 2D US images in the 3D anatomy of the patient to perform the right diagnosis and treatment. The conventional image display on a screen is neither intuitive nor ergonomically friendly in this context. This inconvenient examination may also be associated with work-related musculoskeletal disorders [3]. Hand-eye coordination is another critical skill for sonographers as the gaze is directed to the screen, while the hand needs to adjust the position and orientation of the US probe. An intuitive and straightforward way of visualization could be beneficial, especially for novices, for whom a higher visuo-spatial skill is related to improved US examination outcomes [4].

The augmented reality (AR) glasses HoloLens 2 (Microsoft, Redmond, WA, USA), a head mounted computer with an optical see-through display, may overcome the aforementioned disadvantages by displaying the US images in situ below the US probe–superimposed on the imaged anatomy. This could improve understanding of anatomy and spatial orientation of structures. In addition to imaging, it is possible to overlay important information such as vital signs or tracked surgical instruments during procedures.

Therefore, in this work, we describe a method for tracking a rigid body based on retroreflective spheres by using the integrated depth camera of the AR glasses. Herewith, we demonstrate the first inside-out six degrees of freedom (DoF) tracking approach with HoloLens 2 for passive marker spheres that neither requires additional expensive tracking hardware nor illumination of the spheres. Additionally, the application is able to stream live images from an US station and display them in situ—resulting in an intuitive and realistic visualization. To provide other researchers in this field with an easy-to-set-up tracking approach, we implemented the algorithm with the Unity game engine (Unity Technologies, San Francisco, CA, USA). The code will be available at https://github.com/BIIG-UC3M/IGT-UltrARsound. A further aim of this study is to provide thorough technical details of this approach such as tracking accuracy in a static and dynamic setting, frequency and latency of displayed US images.

### Related work

The idea of using AR to support US examinations was already described in 1996 by Fuchs et al. [5] demonstrating its research interest. Due to its early stage development, the AR glasses weighed nearly 3 *kg*, and the probe was spatially tracked using a mechanical arm. Thus, the whole setup was still far from being usable in daily practice. More than two decades later, the tetherless standalone AR device HoloLens 2, with a weight of 566 g, improved image resolution and self-localization, hand gestures and voice commands for interactions could bring the initial idea closer to the actual clinical application. In situ visualization of US with AR is certainly possible with other state-of-the-art visors [6]; however, in the following section, we want to concentrate on HoloLens as it provides the previously mentioned key features.

#### Tracking with HoloLens

Two approaches for instrument tracking with AR glasses exist. On the one hand, outside-in techniques utilize external sensors (e.g., optical or electromagnetic tracking) placed statically in the room to track the instrument as well as the HoloLens. On the other hand, inside-out techniques perform tracking via cameras and sensors integrated into the AR glasses—thus no additional hardware nor a cumbersome calibration would be necessary. Furthermore, incorporating an external tracking system leads to a more laborious system setup and workflow.

**Outside-in tracking**

A clear advantage of outside-in techniques is the achievable accuracy and precision that certain medical interventions require. However, the use of an external tracking system in combination with HoloLens requires an often cumbersome registration to have a common coordinate system. In many cases, optical tracking systems (OTS) facilitate tracking of medical instruments [7–10]. In this scenario, line of sight between tracked tools (e.g., AR glasses and US probe) and OTS needs to be ensured at all times which limits the possibilities of movement. Electromagnetic tracking, on the other hand, does not have this disadvantage but suffers from interference with metallic parts. Nevertheless, in scenarios such as AR-guided endovascular interventions [11, 12], accurate tracking without line of sight is only provided by this modality.

**Inside-out tracking**

The most common way of tracking surgical tools is to utilize the front-facing central camera for detecting AR markers [13–17]. A general drawback, nonetheless, is that the markers constantly need to point towards the front camera, limiting the movement and rotation of the tracked tool. Additionally, the tracking process depends heavily on light conditions. As an alternative, retroreflective spheres can be used for rigid body tracking with HoloLens. Kunz et al. [18] showed the feasibility of this approach with HoloLens 1 by either using the integrated depth camera or two of the four front facing cameras via triangulation. However, tracking with the front-facing cameras requires placing an IR light emitter on the HoloLens. Additionally, this work only evaluated relative translations (three DoF) of the spheres, thus no conclusions can be made regarding the six DoF rigid body tracking. A recent proposal by Gsaxner et al. [19] showed promising results of rigid body tracking with retroreflective markers via triangulation, obtaining an accuracy of 1.70 mm/1.11$^\circ$ with an extended Kalman filter. However, this approach again involves a supplementary light source on HoloLens to illuminate the spheres which might impair the mobility of the AR glasses. Considering the previous approaches, in this work, we want to exploit the advantage of tracking retroreflective spheres with an inside-out approach, namely less light dependency and no need for external expensive tracking systems, without the necessity of additional hardware to illuminate the spheres.

#### Ultrasound visualization with HoloLens

Only a few studies have investigated the combination of US imaging and HoloLens. Some of them rely on outside-in approaches to track the US probe and display the image [8, 9, 20]. Existing works utilizing inside-out approaches are on the other hand performed using an AR marker [11, 21, 22]. To acquire and stream images from the US station to HoloLens, one can either use a frame grabber at the display output of the US station or, if available, a software development kit (SDK) providing access to the image data. Nguyen et al. [22] followed the latter approach and then downsampled the images to 100 $\times$ 100 pixels to send them as a single UDP package. However, this resizing comes with an image quality loss. On the other hand, Rüger et al. [9] and Kuzhagaliyev et al. [8] grabbed the screen output of the US stations and sent the trimmed images to HoloLens. Here again, depending on the image output, an image quality loss is possible. This loss of quality may become relevant when the US image in HoloLens is enlarged ex situ to see finer structures. Our approach overcomes these disadvantages by streaming US images in original image quality with minimal latency and a commonly used open-source network communication interface (OpenIGTLink).

## Materials and methods

The developed application runs directly on HoloLens 2 and was built with Unity 2019.4.22. The research mode was incorporated within Unity [23] allowing access to the following sensors: four grayscale cameras, the inertial measuring unit and the depth camera which operates in two modes for different distances—each one providing a depth image and an active brightness (AB) image [24]. On top, OpenCV was integrated within Unity for image processing tasks. The depth camera offers two different modes, whereas in this work, only the AHAT (Articulated HAnd Tracking) mode was used because it provides more frames per second and the maximum distance of 1 m is sufficient for tracking a handheld US probe. The depth and AB image of the AHAT mode have a resolution of 512 $\times$ 512 pixels with 16 bits per pixel acquired at 45 frames per second. To track the pose of our rigid body composed of retroreflective spheres, the workflow described in detail in Sects. 2.1 and 2.2 consists of the following steps: (1) Measure the position of each sphere with respect to our world coordinate system using the depth camera. (2) Apply some filtering to reduce the noisy measurements of the depth camera. (3) Calculate the pose based on the resulting positions.

For US acquisitions, we used the portable system MicrUs from TELEMED (Vilnius, Lithuania) in combination with the linear US probe L12-5L40S-3. This system offers access to the US images without requiring a frame grabber. In Sect. 2.3, we describe the network communication interface and the registration approach required to find the spatial transformation from the tracked marker to the origin of the image.

### Retroreflective sphere tracking

**Fig. 1:**
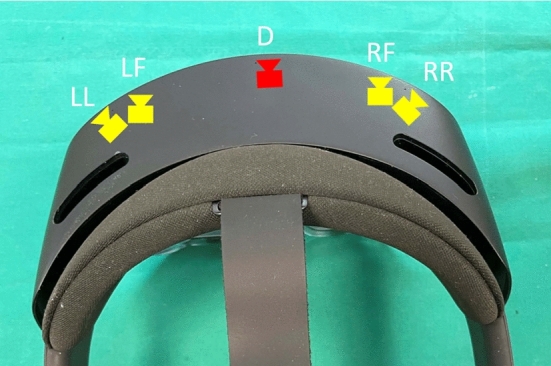
Top view of HoloLens 2, the different visible light cameras (yellow), and the depth camera (red). The research mode provides for each acquired frame the pose of the left-front (LF) visible light camera. The extrinsic transform from the left-front camera to the depth camera can also be accessed via the research mode

In order to compute ${}^{W}{{{\textbf {s}}}} = \left[ x,y,z,1\right] ^\text {T}$, which is the surface position of a retroreflective sphere with respect to a world coordinate system *W*, the following equation is applied:

1 $$\begin{aligned} {}^{W}{{{\textbf {s}}}}={}^{W}{{{\textbf {T}}}}_L \, {}^{L}{{{\textbf {T}}}}_D \, {}^{D}{{{\textbf {s}}}} \end{aligned}$$

where ${}^{W}{{{\textbf {T}}}}_L \in {\mathbb {R}}^{4 \times 4}$ is the pose of the left-front camera of HoloLens 2 (Fig. 1) with respect to our world coordinate system, ${}^{L}{{{\textbf {T}}}}_D$ is the extrinsic camera matrix of the depth camera which can be accessed via the research mode and ${}^{D}{{{\textbf {s}}}}$ is the surface position of a sphere with respect to the depth camera coordinate system. ${}^{W}{{{\textbf {T}}}}_L$ is provided by the research mode for each frame, while the origin of the world coordinate system is placed at the device’s pose at application start. To track the retroreflective spheres and hereby the marker in the camera space, one first needs to detect the spheres in the AB image of the AHAT mode, which then allows finding the corresponding points in the depth image of the AHAT mode.

**Fig. 2:**
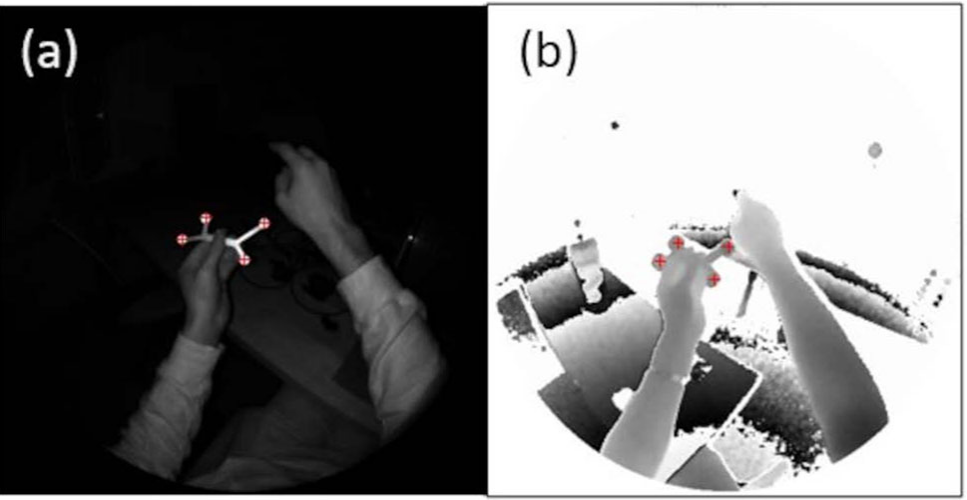
**a** AB image with the detected centers of the spheres (red). **b** The corresponding depth image. The corresponding pixel values are used to calculate the position of the spheres using a lookup table

A blob detection algorithm provided by OpenCV is utilized to detect the spheres in the AB image (Fig. 2a). For each of the $i=1\dots 4$ spheres in the image, the method returns the pixel coordinates ${{\textbf {p}}}_{i}$ corresponding to the center of the circular image of the sphere. Then, the corresponding pixel value $d_{{{\textbf {p}}}}$ in the depth image (Fig. 2b) is multiplied by the appropriate vector from a static lookup table ${{\textbf {L}}}\in {\mathbb {R}}^{512\times 512\times 3}$ (also accessible via the research mode) to compute the initial guess of the surface sphere position ${}^{D}{{{\textbf {s}}}} = [x,y,z]^T$ in the depth camera coordinate system:

2 $$\begin{aligned} {}^{D}{{{\textbf {s}}}} = \begin{bmatrix} x\\ y\\ z \end{bmatrix} =d_{{{\textbf {p}}}} \; {{\textbf {L}}}({{\textbf {p}}}) = d_{{{\textbf {p}}}} \, \begin{bmatrix} u_{}\\ v_{}\\ w_{}\end{bmatrix} \end{aligned}$$

where $\left[ u,v,w\right] ^\text {T}$ is the entry of the lookup table corresponding to the image position ${{\textbf {p}}}$.

As our approach does not directly allow tracking the center position of the spheres, but just a point on the surface, a correction vector is added to the surface position ${}^{W}{{{\textbf {s}}}}$. The direction and length of this vector can be calculated by subtracting the position of the measured surface point ${}^{W}{{{\textbf {s}}}}$ and the depth camera ${}^{W}{{{\textbf {c}}}_d}$ with a length equal to the sphere radius ${r}_s$. Thus, the final center sphere position ${{\textbf {s}}}_c$ is calculated as:

3 $$\begin{aligned} {}^{W}{{{\textbf {s}}}}_c = {}^{W}{{{\textbf {s}}}} +\frac{{}^{W}{{{\textbf {s}}}} - {}^{W}{{{\textbf {c}}}}_d}{\mid \mid {}^{W}{{{\textbf {s}}}} - {}^{W}{{{\textbf {c}}}_d} \mid \mid } r_s \end{aligned}$$

**Position estimation using a Kalman filter**

A Kalman filter [25] is a mathematical method for the iterative estimation of parameters describing system states on the basis of potentially erroneous observations. As the measurements of the depth camera of HoloLens 2, which is hardware-wise the same as the Azure Kinect camera, show a noisy behavior [26], we decided to implement a Kalman filter to reduce the influence of erroneous measurements. To this end, a 3D constant acceleration model served as underlying motion model for each measured sphere position. We determine the optimal values of the process noise covariance $\sigma _Q$ and measurement noise covariance $\sigma _R$ through testing on a prerecorded sequence obtaining $\sigma _Q = 0.0001$ and $\sigma _R = 0.001$ for the static setting. In a dynamic environment, the optimal values were $\sigma _Q = 0.0001$ and $\sigma _R = 0.0001$.

### Marker pose calculation

The four sphere center positions ${}^{W}{{{\textbf {s}}}}_{c,i}$ in the world coordinate system can be used to calculate the pose ${}^{W}{{{\textbf {T}}}}_M$. Therefore, to avoid an ambiguous identification of the order, the relative distances between the spheres need to be unique. The 3D coordinates of the sphere centers are expressed in a common reference frame whose origin can be placed at a desired position and orientation. In this work, we used an OTS, more precisely Polaris Spectra from NDI, to measure the distances and create the origin of our reference frame including the positions ${}^{RF}{{{\textbf {s}}}}_{c,i}$ of the sphere centers. The tool definition is performed beforehand and provided to the tracking algorithm. Within the tracking algorithm and for each cycle, we first measure the distances of the spheres to each other to identify corresponding points and then perform a landmark-based registration [27] using ${}^{RF}{{{\textbf {s}}}}_{c,i}$ and ${}^{W}{{{\textbf {s}}}}_{c,i}$ to compute ${}^{W}{{{\textbf {T}}}}_M$.

### Ultrasound streaming and calibration

**Fig. 3:**
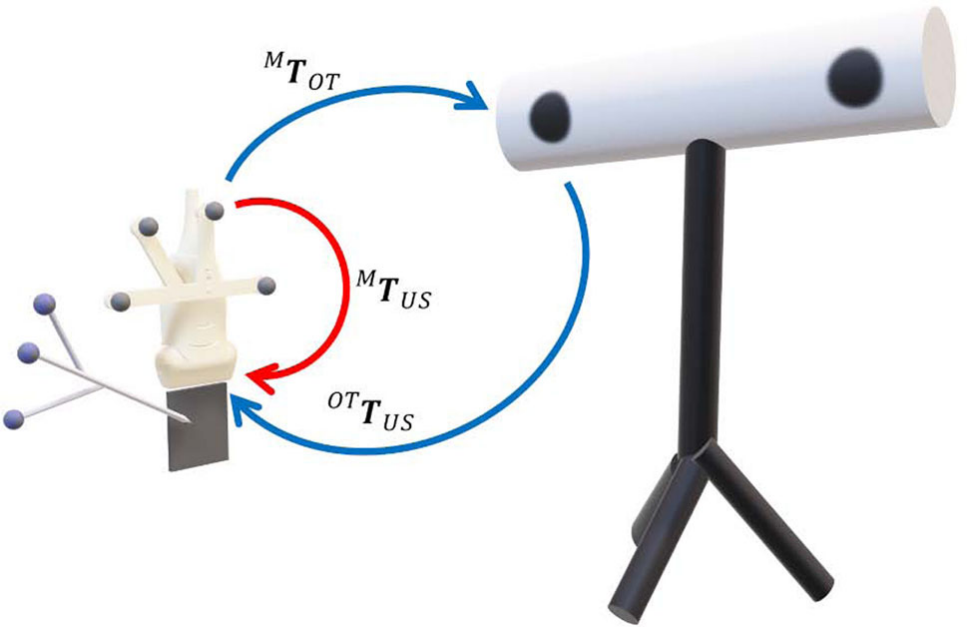
Transformations (arrows) available and calculated for the spatial calibration between the US marker and the US image. The measured positions of the stylus tip in the US images and tracking space allow the calculation of ${}^{\mathrm{OT}}{{{\textbf {T}}}}_{US}$ and thereafter close the loop to compute ${}^{M}{{{\textbf {T}}}}_{US}$ (red arrow)

The US images were sent via the open-source-toolkit PLUS [28] from the computer to the Unity application running on HoloLens 2. As no library exists in Unity supporting the OpenIGTLink protocol [29] used by PLUS, we developed a custom-made client side. In order to display the US images in situ on the AR glasses, it is necessary to find the spatial transformation from the tracked marker to the origin of the image ${}^{M}{{{\textbf {T}}}}_{US}$. To this end, we used a phantom-free approach [30] that requires an external OTS. We opted for this approach as it shows better results than the commonly used N-wire phantom [31]. For the calibration, we recorded the static pose of the optically tracked US probe marker ${}^{\mathrm{OT}}{{{\textbf {T}}}}_{M}$ immersed in saltwater. Using a tracked and pivot-calibrated stylus, we successively measured eight stylus tip positions in the US beam (Fig. 3). Identifying the positions of the stylus tip in the eight US images and simultaneously in the OTS space allows for the calculation of the transformation of the US image in the OTS coordinate space ${}^{\mathrm{OT}}{{{\textbf {T}}}}_{US}$ by means of a landmark-based registration [27]. Finally, we can compute the desired transformation by closing the transformation loop with:

4 $$\begin{aligned} {}^{M}{{{\textbf {T}}}}_{US}={}^{M}{{{\textbf {T}}}}_{OT} \, {}^{\mathrm{OT}}{{{\textbf {T}}}}_{US} \end{aligned}$$

The 3D models for the attachment of the markers used in this work were retrieved from PLUS [32].

### Evaluation

Apart from testing the feasibility, the aim of this work was to perform a technical evaluation of the proposed approach. To this end, we measured the tracking accuracy of the sphere’s positions and the pose estimation in a static scenario. To also evaluate the system in a realistic environment, the pose tracking accuracy was measured in a dynamic scenario. Additionally, we measured the time duration of one tracking cycle and the latency of the displayed US images on HoloLens 2.

#### Retroreflective sphere tracking

**Fig. 4:**
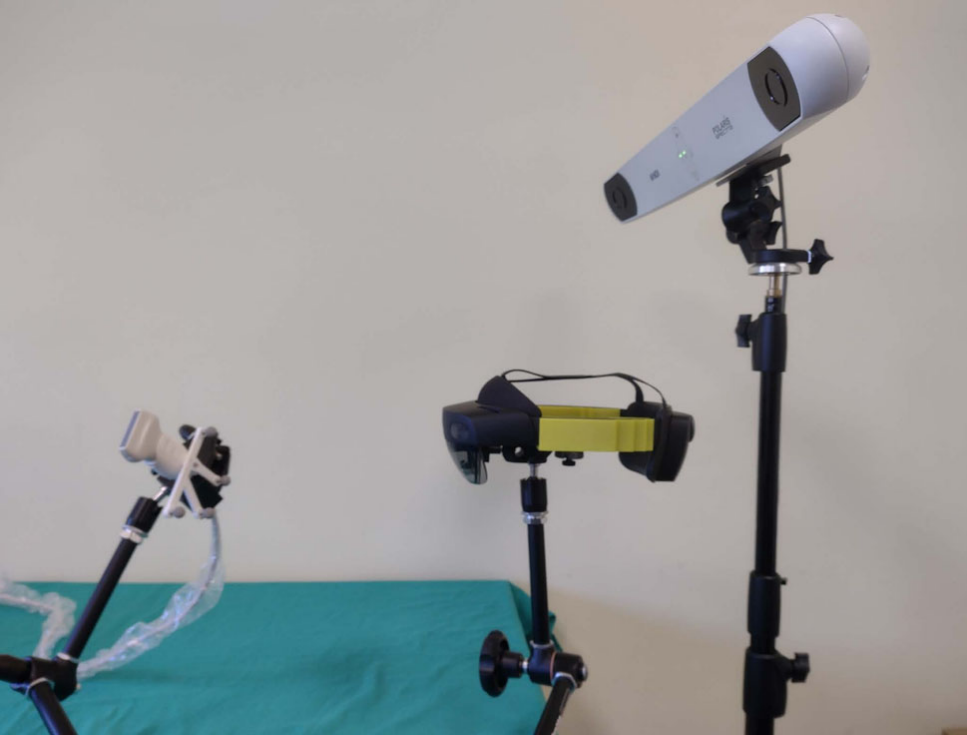
Setup for the static tracking accuracy evaluation. HoloLens 2 and the OTS are fixed, while the US probe with its marker is moved to 20 different poses for measurement acquisition

To quantify the accuracy of our tracking method in a static scenario, we compared relative movements of the marker measured with the AR glasses and, as a ground truth, the highly accurate OTS (Fig. 4). Twenty different poses of the marker in a range from approximately 0.3 to 1 *m* were recorded acquiring 30 samples for each pose. Thereafter, for each sample and each pose, the relative distance of each sphere to the corresponding sphere in the first pose was computed and compared to the value obtained with OTS. For example, to calculate the distance error $d_e$ between the second and first pose for one sphere, we compute:

5 $$\begin{aligned} {d}_{e} =\mid \mid {}^{W}{{{\textbf {s}}}_{c,p_2}} -{}^{W}{{{\textbf {s}}}_{c,p_1}} \mid \mid - \mid \mid {}^{\mathrm{OT}}{{{\textbf {s}}}_{c,p_2}} -{}^{\mathrm{OT}}{{{\textbf {s}}}_{c,p_1}} \mid \mid \end{aligned}$$

where ${}^{\mathrm{OT}}{{{\textbf {s}}}_{c,p_1}}$ is a sphere center position for the first pose in the OTS coordinate system. To additionally evaluate the actual marker pose tracking accuracy, the pose change of each marker, which is based on the four sphere positions, was also analyzed. Here, we compared the relative translational and rotational movements between poses measured with HoloLens 2 and the OTS. Again, for example, to measure the translation error ${{\textbf {t}}}_{e}$ and rotational error ${{\textbf {r}}}_{e}$ between the second and first pose, we first measure the pose error ${{\textbf {T}}}_{e}$:

6 $$\begin{aligned} {{\textbf {T}}}_{e} = (({}^{W}{{{\textbf {T}}}_{M,p_1}})^{-1} \, {}^{W}{{{\textbf {T}}}_{M,p_2}})^{-1}\,(({}^{\mathrm{OT}}{{{\textbf {T}}}_{M,p_1})^{-1}}\,{}^{\mathrm{OT}}{{{\textbf {T}}}_{M,p_2}}) \end{aligned}$$

which we use to extract ${{\textbf {t}}}_{e}$ and ${{\textbf {r}}}_{e}$ in Euler angles.

**Fig. 5:**
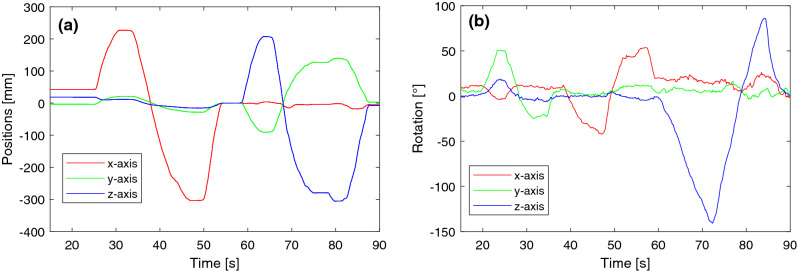
Trajectories obtained with the OTS for the dynamic analysis. **a** Relative positions and **b** relative rotations for each axis with the respect to the initial pose

For the dynamic scenario on the other hand, we acquired a trajectory of our marker with the statically placed AR glasses and the OTS (trajectory illustrated in Fig. 5). As line of sight of the spheres is necessary, the possible rotation around the x- and y-axis was limited. For the temporal calibration between the measured trajectories of HoloLens 2 and the tracking system, we acquired a sinusoidal trajectory before the actual acquisition. Thereafter, the sinusoidal signals were downsampled to have matching frequencies. Finally, the maximum position of the cross-correlation of both signals allows to calculate the time shift between both measurements. As in the static scenario, we compute ${{\textbf {t}}}_{e}$ and ${{\textbf {r}}}_{e}$ with the initial pose as our reference. To measure the tracking frequency, the time interval of 500 tracking cycles was obtained.

**Fig. 6:**
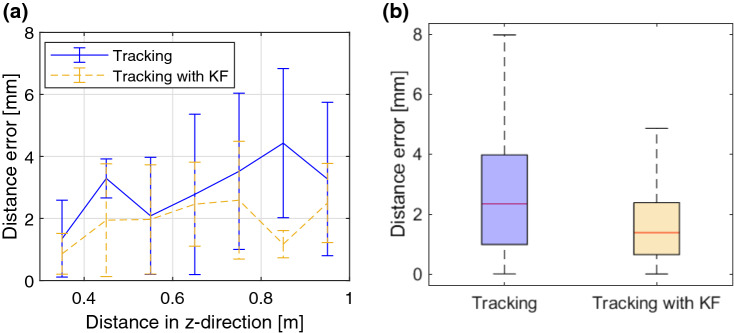
Results of the accuracy evaluation for position tracking with HoloLens 2. **a** Mean and standard deviation of $d_{e,\mathrm{RMS}}$ versus the distance to the depth camera. **b** Distribution (median, 25th and 75th percentile, minimal and maximal errors) of $d_{e,\mathrm{RMS}}$ for tracking with and without Kalman filter

#### Ultrasound streaming

Apart from a correct in situ placement of the US image, it is essential that the latency of displayed US images on HoloLens 2 is low enough for a real-time perception. To measure the latency, a slow motion video with 120 Hz is recorded which results in a temporal resolution of 8 ms. The video is recorded through the lenses of the AR glasses such that the virtual canvas including the US image and the US image on the real screen are visible. To measure the delay, the depth of the US image is changed and the frames are counted until the change becomes visible on the virtual canvas. For the evaluation, the latency was measured 30 times. Images with a matrix size of 512 $\times$ 512 pixels and 8 bits per pixel were sent. Note that the image matrix size does not change even if the depth is modified.

## Results

Following, the evaluation results of the tracking accuracy, tracking frequency and the US streaming latency are described. Within the tracking accuracy evaluation, we first present the results of the static and dynamic scenario separately and then compare them .

### Retroreflective sphere tracking

We compared the accuracy of the spheres position tracking with and without the implemented Kalman filter. The mean distance error and standard deviation without filter was $d_{e,\mathrm{RMS}} = 3.27 \pm 1.90$ mm. On the other hand, the Kalman filter lowered the distance error to $d_{e,\mathrm{RMS}} = 2.23 \pm 1.38$ mm (Fig. 6b). As shown in Fig. 6a, the error depends on the distance to the depth camera and increases roughly by 1.5 mm between 0.3 and 1 m. For all distances except one, the standard deviation decreases once the filter is applied.

**Fig. 7:**
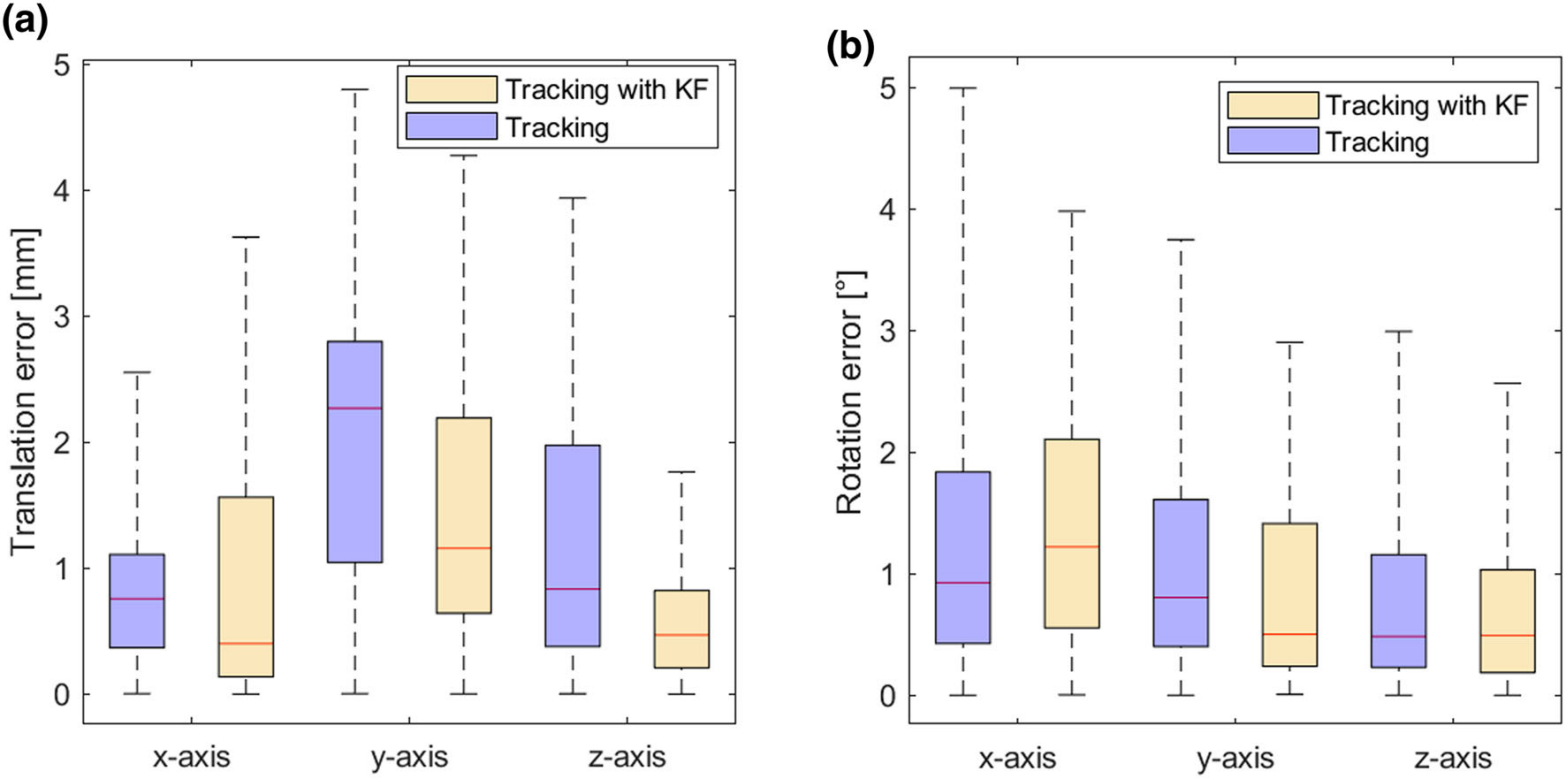
Results of the accuracy evaluation for pose tracking with HoloLens 2 in a static scenario. **a** The distribution of the translational error and **b** the distribution of the rotational error with and without Kalman filter

In a second step, we compared the accuracy of the pose tracking with and without Kalman filter for a static scenario. Again, only the relative movements to the first obtained pose are evaluated. In Fig. 7, ${{\textbf {t}}}_{e,\mathrm{RMS}}$ and ${{\textbf {r}}}_{e,\mathrm{RMS}}$ are visualized for each axis. When looking at ${{\textbf {t}}}_{e,\mathrm{RMS}}$, we see that applying a Kalman filter lowers the median error for each axis. Except for the *x*-axis, the 75th percentile and the maximum error are lower when using a Kalman filter. For the rotational error ${{\textbf {r}}}_{e,\mathrm{RMS}}$, only a slight improvement can be achieved with the filter. In all cases, the median is close to or lower than 1.

**Fig. 8:**
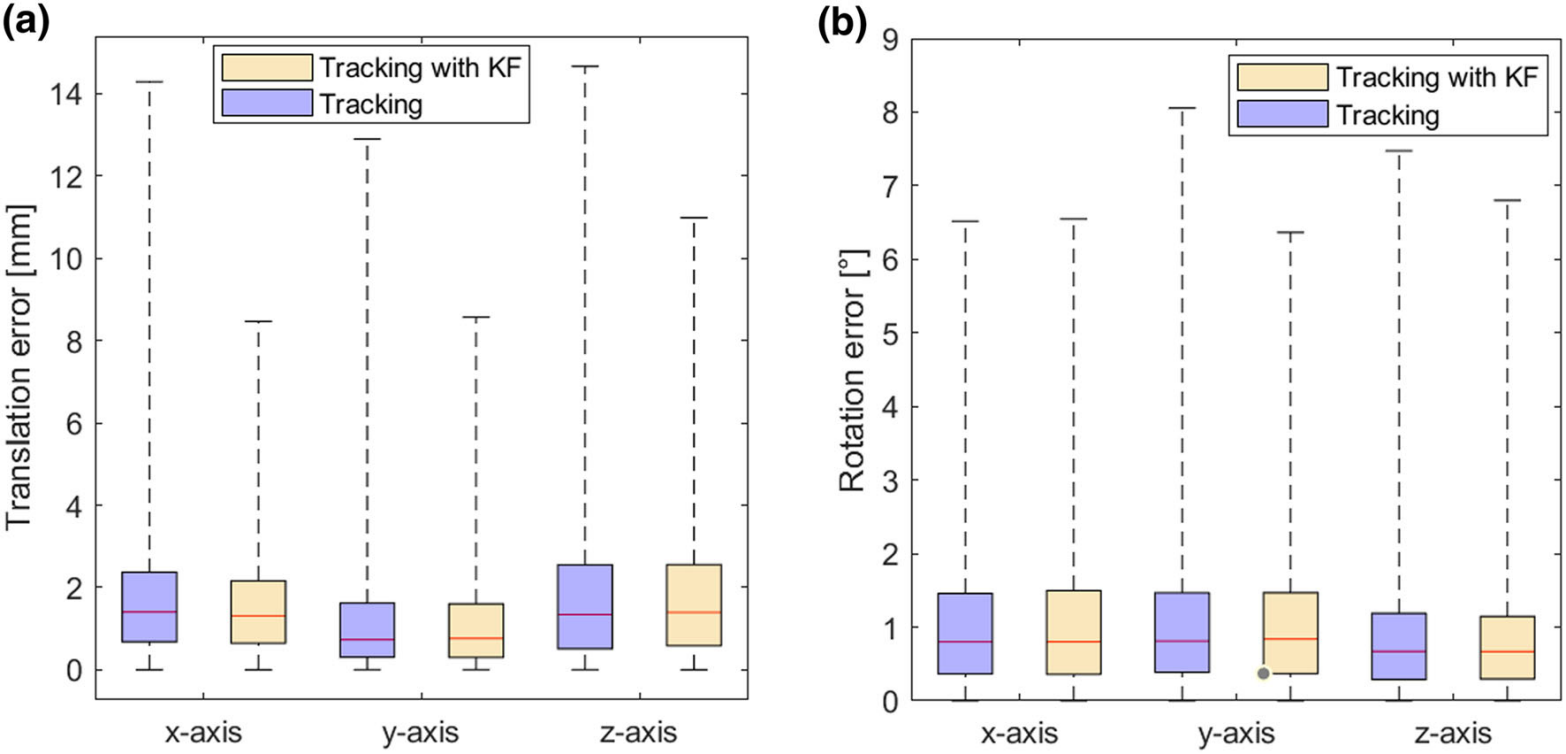
Results of the accuracy evaluation for pose tracking with HoloLens 2 in a dynamic scenario. **a** The distribution of the translational error with and without Kalman filter. **b** The distribution of the rotational error for each axis with and without Kalman filter

In a third step, we compared the accuracy of the pose tracking in a dynamic scenario. Figure 8 shows the distribution of ${{\textbf {t}}}_{e,\mathrm{RMS}}$ and ${{\textbf {r}}}_{e,\mathrm{RMS}}$ for each axis. We can clearly see that in a dynamic scenario, we obtain errors higher than roughly 3 mm for one fourth of the measurements with maximum errors of around 15 mm. On the other side, the median values are below 2 mm . The Kalman filter is able to lower the higher errors to a maximum value of  10 mm, while the median values are similar in both cases. Overall, we see a similar behavior for the rotational error ${{\textbf {r}}}_{e,\mathrm{RMS}}$, namely partially higher errors with low median errors. In this case, however, the Kalman filter brings only a slight improvement regarding the accuracy.

Table 1 shows the statistics of the errors obtained in both scenarios for a comparative analysis. Note that, to this end, we computed the magnitude of ${{\textbf {t}}}_{e}$ and ${{\textbf {r}}}_{e}$ which is a valid option in our case [33]. When looking at $\mid \mid {{\textbf {t}}}_{e} \mid \mid$, we see that the median only slightly increases without any filter. The RMS value decreases by roughly 1 mm when applying the filter for both scenarios. For $\mid \mid {{\textbf {r}}}_{e} \mid \mid$, the median as well as the RMS values remain similar for all cases. To summarize, we see that the Kalman filter improves the accuracy regarding the translation measurements, while it does not have a noticeable effect on the rotational values.

Finally, the average time and standard deviation for one tracking cycle was 50.64 ± 7.11 ms resulting in a frequency of around 20 Hz.

### Ultrasound streaming

**Fig. 9:**
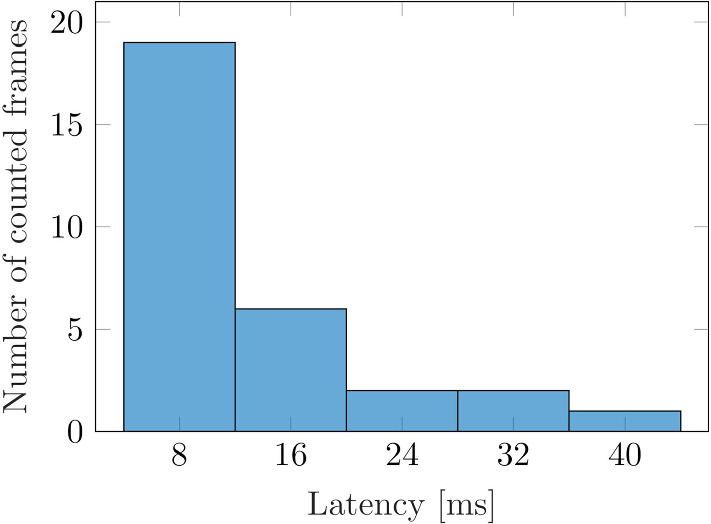
Results of the latency measurement of displayed US images on HoloLens. A careful examination of the slow-motion video with 120 Hz allows to count the frames until a change in the US image is visible on HoloLens 2

**Fig. 10:**
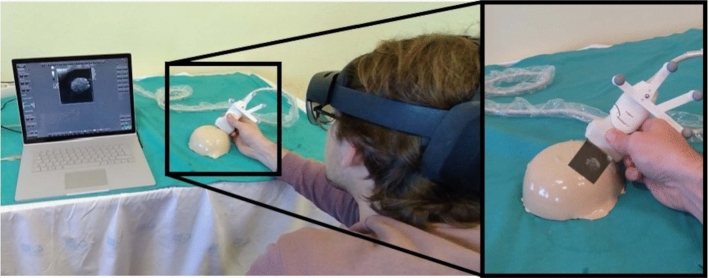
HoloLens 2 user holding the US probe with the attached retroreflective spheres during a breast phantom acquisition, showing the US image on the computer screen (left). The view through the AR glasses enables to see the image in situ (right)

83% of the frames had a delay of 2 or less frames and thus a maximum delay of 16 *ms* (Fig. 9). Figure 10 shows the application running on HoloLens 2. The US image of a breast phantom is visible on the computer screen, while the user can see the image in situ through the AR glasses.

**Table 1:** Error statistics of $\mid \mid {{\textbf {t}}}_{e}\mid \mid$ and $\mid \mid {{\textbf {r}}}_{e} \mid \mid$ for dynamic and static scenarios with and without Kalman filter

		Min	25th p	Median	75th p	Max	RMS
		Translation error [mm]					
Static	Tracking only	0.03	1.73	2.71	3.50	5.93	3.07
	With Kalman filter	0.02	1.24	1.98	2.58	4.70	2.14
Dynamic	Tracking only	0.15	2.02	3.01	4.42	17.58	4.79
	With Kalman filter	0.08	1.96	2.81	3.79	16.15	3.76
		Rotation error [${}^\circ \!$]					
Static	Tracking only	0.10	1.21	2.01	2.74	4.32	2.24
	With Kalman filter	0.01	1.11	1.81	2.67	4.11	2.16
Dynamic	Tracking only	0.19	1.17	1.75	2.49	8.51	2.25
	With Kalman filter	0.23	1.18	1.70	2.46	10.07	2.19

## Discussion

US requires skilled and experienced sonographers for a correct and accurate diagnosis. This is partially explained by the mental workload of aligning small cross-sectional 2D images in the 3D anatomy of the patient. Our application UltrARsound overcomes this disadvantage by displaying live US images in situ using the AR glasses HoloLens 2. In this work, we describe an approach to track the US probe with retroreflective spheres with an inside-out technique, utilizing solely the depth camera of HoloLens 2. Accordingly, no expensive additional hardware is necessary. Further, we show how to stream US images from an US station with minimal latency. For a final evaluation, we perform a technical characterization of the application by measuring spatial tracking accuracy and latency of displayed US images.

We measured the accuracy by comparing relative movements obtained with HoloLens 2 and a high accuracy OTS. The results show that the position tracking accuracy improves when applying a Kalman filter. Not only does the median of $d_{e,\mathrm{RMS}}$ decrease but so do the 75th percentile and maximum values. Consequently, we also see a decrease in ${{\textbf {t}}}_{e,\mathrm{RMS}}$ for each axis. The rotation error, otherwise, remains similar. A possible explanation is that if we measure the same incorrect position shift for each sphere, the translation error increases, while the rotation error stays the same. Indeed, our measured errors align with previously published works on the accuracy of the depth camera [26] in which errors between 1.5 to 2.5 mm are stated for distances of 0.5 to 1 m in the binned wide mode. A study published by Microsoft [34] states slightly lower values (1–2 mm). However, they do not differentiate between different modes which makes a comparison difficult. To conclude, we assume that a majority of the measured error in a static setting is due to hardware limitations. Nevertheless, the following software-related steps could improve the results. It is assumed that the static lookup table ${{\textbf {L}}}$ used to calculate the positions is perfect. A re-calibration might lower $d_{e,\mathrm{RMS}}$ and therefore ${{\textbf {T}}}_{e}$. Additionally, our approach assumes that measurement vector of the Kalman filter consists of the calculated positions in all three dimensions. Another option that could potentially further improve the results is to use the measured blob location ${{\textbf {p}}}$ in the AB image as measurement vector instead. However, to calculate the predicted measurement from the state prediction, one would need to know the inverse version ${{\textbf {L}}}$ which is not available to our knowledge. Therefore, we relied on a simplified model for our Kalman filter.

Regarding the dynamic measurements, it is to be expected that the errors increase. However, it is spiking that a quarter of the measurements (Fig. 8) range from 4 to 15 mm. In this case, as no literature exists that investigates the accuracy of the depth camera in a dynamic scenario, it is difficult to determine if the error is hardware- or software-related. Applying a Kalman filter improves the accuracy regarding the translation measurements in both settings. To obtain the best results, it was necessary to apply different values of the noise covariance and measurement noise covariance for the static and the dynamic scenario, respectively.

Gsaxner et al. [19] showed slightly lower errors for translation and rotation with 1.70 mm and 1.11$^\circ$ in a static setup. In a dynamic setting, their approach outperforms ours with 1.90 mm and 1.18$^\circ$. However, we would like to note that our approach does not need an additional light source to illuminate the retroreflective spheres. Additionally, Gsaxner et al. [19] compared absolute positions and rotations of HoloLens 2 and an OTS in the same coordinate system which was achieved by means of a hand-eye-calibration. Thus, a direct comparison between the two approaches is challenging. We decided to compare only relative movements of the marker as it is not clear how much the hand-eye-calibration influences the measured error.

With the current arrangement of the spheres, it is inevitable that there are some blind spots such that the rigid body cannot be tracked. For the proposed application, we assume that the possible rotations are sufficient as the sonographer will not look at the US image turned by 90$^\circ$ so that only the edge of the image is visible. It is possible to add more spheres to the rigid body which would allow to track the body with less or even no movement constraints. However, this would impair the handiness and maneuverability of the US probe with the attached rigid body. Therefore, we decided to use the rigid body provided by the PLUS library and accept to lose some movement freedom.

Overall, the tracking accuracy reported here is promising for training cases in ultrasound diagnostics in which a sub-millimeter accurate overlay of real and imaged anatomy is not required. It is worth mentioning that the accuracy might be lower in a real scenario in which the HoloLens will also move. In this work, the AR glasses are placed statically throughout the measurements. Thus, the error of the self-localization of HoloLens is not taken into account. A previous study showed that the error of the self-localization may be up to 3 cm in a room-scale environment [35]. Consequently, the accuracy of our approach may be greatly influenced by a movement of the HoloLens.

Using the proposed approach, a tracking frequency of 20 Hz was achieved. We believe that tracking frequency should ideally be $\ge$ 60 Hz to match the frame rate of HoloLens 2 and provide a smooth visualization. Therefore, future work will consist of an analysis of the algorithm to identify the most time-consuming parts. An initial improvement might be achieved by performing the blob detection only in a region of interest defined by the previous found locations of the blobs.

Low latencies are crucial for in situ visualization of US images especially when guiding interventions such as biopsies. In this work, the latency of displayed US images was sufficiently low for a real-time perception with a majority of frames being < 16 ms delayed, proving that our approach is faster than the 80 ms reported by Ngyuen et al. [22]. García-Vázquez et al. [11] stated a latency of 259 ms for US volumes, correspondingly a significantly higher amount of data was transmitted which makes a comparison difficult. Other studies mentioned in Sect. 1.1.2 did not measure any latencies. The development of the client-side based on the OpenIGTLink protocol additionally allows connecting any other US station that supports streaming via the PLUS toolkit. Nevertheless, the US calibration needs to be performed for each new US probe.

## Summary

This work presents an approach to spatially track retroreflective spheres with HoloLens 2 which in turn allows to track the position and orientation of an US probe with attached spheres. For in situ visualization, live US images are streamed from the US station to the AR glasses wirelessly. The inside-out tracking is performed through the depth camera of HoloLens 2. Accordingly, neither an external tracking system nor illumination of the spheres with additional hardware is required and, thus, it provides a cost and space efficient alternative to other approaches. The achieved accuracy is promising for diagnostic US examinations and partially for image-guided therapies in which no sub-millimeter accuracy is necessary. We believe that sub-millimeter accuracy may be achievable if both the depth camera and the stereo setup of the visible light cameras are taken into account, and both measurements are inputs to the Kalman filter.

Overall, our open-source application UltrARsound may allow sonographers to learn and perform US examinations faster and more effectively by providing an intuitive and ergonomically friendly in situ visualization of the 2D US image.
